# Intra-lake response of Arcellinida (testate lobose amoebae) to gold mining-derived arsenic contamination in northern Canada: Implications for environmental monitoring

**DOI:** 10.7717/peerj.9054

**Published:** 2020-05-04

**Authors:** Nawaf A. Nasser, R. Timothy Patterson, Jennifer M. Galloway, Hendrik Falck

**Affiliations:** 1 Ottawa-Carleton Geoscience Centre and Department of Earth Sciences, Carleton University, Ottawa, ON, Canada; 2 Geological Survey of Canada (GSC)/Commission géologique du Canada, Natural Resources Canada (NRCan)/Ressources naturelles Canada (RNCan), Calgary, AB, Canada; 3 Aarhus Institute of Advanced Studies (AIAS), Aarhus University, Aarhus, Denmark; 4 Northwest Territories Geological Survey, Yellowknife, NT, Canada

**Keywords:** Bio-monitoring, Lake sediments, Legacy contamination, Intra-lake sampling, Multivariate analysis, Northwest Territories

## Abstract

Arcellinida (testate lobose amoebae) were examined from 40 near-surface sediment samples (top 0.5 cm) from two lakes impacted by arsenic (As) contamination associated with legacy gold mining in subarctic Canada. The objectives of the study are two folds: quantify the response of Arcellinida to intra-lake variability of As and other physicochemical controls, and evaluate whether the impact of As contamination derived from two former gold mines, Giant Mine (1938–2004) and Tundra Mine (1964–1968 and 1983–1986), on the Arcellinida distribution in both lakes is comparable or different. Cluster analysis and nonmetric multidimensional scaling (NMDS) were used to identify Arcellinida assemblages in both lakes, and redundancy analysis (RDA) was used to quantify the relationship between the assemblages, As, and other geochemical and sedimentological parameters. Cluster analysis and NMDS revealed four distinct arcellinidan assemblages in Frame Lake (assemblages 1–4) and two in Hambone Lake (assemblages 5 and 6): (1) Extreme As Contamination (EAC) Assemblage; (2) High calcium (HC) Assemblage; (3) Moderate As Contamination (MAC) assemblages; (4) High Nutrients (HN) Assemblage; (5) High Diversity (HD) Assemblage; and (6) *Centropyxis aculeata* (CA) Assemblage. RDA analysis showed that the faunal structure of the Frame Lake assemblages was controlled by five variables that explained 43.2% of the total faunal variance, with As (15.8%), Olsen phosphorous (Olsen-P; 10.5%), and Ca (9.5%) being the most statistically significant (*p* < 0.004). Stress-tolerant arcellinidan taxa were associated with elevated As concentrations (e.g., EAC and MAC; As concentrations range = 145.1–1336.6 mg kg^−1^; *n* = 11 samples), while stress-sensitive taxa thrived in relatively healthier assemblages found in substrates with lower As concentrations and higher concentrations of nutrients, such as Olsen-P and Ca (e.g., HC and HM; As concentrations range = 151.1–492.3 mg kg^−1^; *n* = 14 samples). In contrast, the impact of As on the arcellinidan distribution was not statistically significant in Hambone Lake (7.6%; *p*-value = 0.152), where the proportion of silt (24.4%; *p*-value = 0.005) and loss-on-ignition-determined minerogenic content (18.5%; *p*-value = 0.021) explained a higher proportion of the total faunal variance (58.4%). However, a notable decrease in arcellinidan species richness and abundance and increase in the proportions of stress-tolerant fauna near Hambone Lake’s outlet (e.g., CA samples) is consistent with a spatial gradient of higher sedimentary As concentration near the outlet, and suggests a lasting, albeit weak, As influence on Arcellinida distribution in the lake. We interpret differences in the influence of sedimentary As concentration on Arcellinida to differences in the predominant As mineralogy in each lake, which is in turn influenced by differences in ore-processing at the former Giant (roasting) and Tundra mines (free-milling).

## Introduction

**Figure 1:**
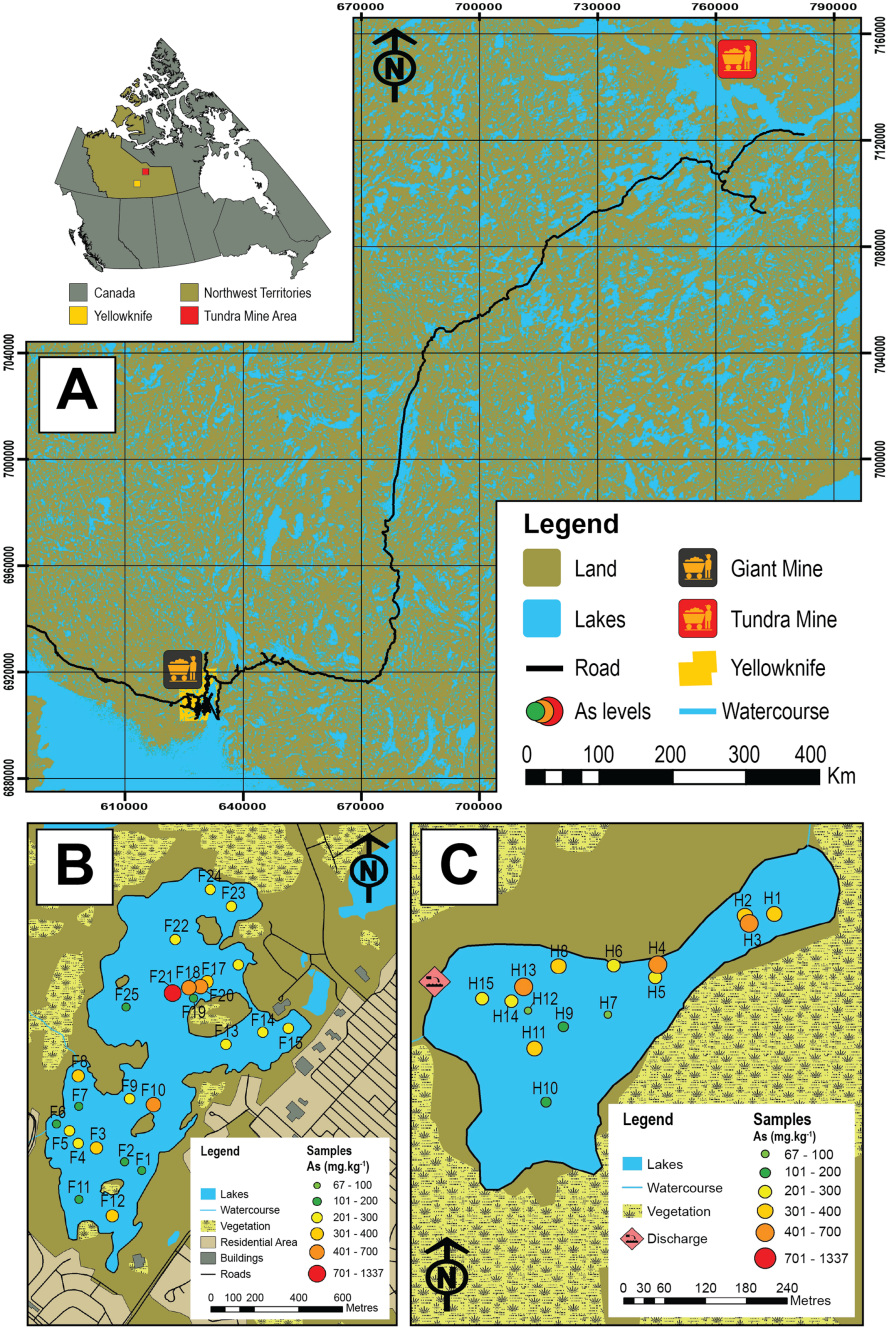
Map of the study area and sampled lakes. (A) A map showing the study area in the central Northwest Territories, Canada and the locations of the historic Giant (black and yellow icon) and Tundra mines (red and yellow icon). (B) A map showing Frame Lake and the location of the 25 sampling stations. (C) A map showing Hambone Lake and the location of the 15 sampling stations. The colour-coded and graduated circles reflect the As concentration at each sampling station.

The mineral mining sector in northern Canada has been a substantial driver of the nation’s economic growth, with a production value of $65 billion since 1974 (Natural Resources Canada, 2019). While highly profitable, many northern mines operated before the concept of sustainable development was introduced in 1987 (World Commission on Environment and Development (WCED), 1987). As a result, several mining operations introduced considerable levels of contaminants, mostly heavy metals, to surrounding environments and this poses a risk to environmental and human health (INAC, 2009; Silke, 2009). Gold mining is a primary anthropogenic source of arsenic (As) contamination in the Northwest Territories (NT; Palmer et al., 2015, 2019; Galloway et al., 2015, 2018; Nasser et al., 2016; Miller et al., 2019). Orogenic greenstone belts in the NT (e.g., Yellowknife and Courageous Lake Greenstone Belts) host large gold deposits that are closely associated with As-bearing sulfides (e.g., arsenopyrite). Ore-processing at several gold mines across the NT, especially Giant Mine in the Yellowknife area (1938–2004; Fig. 1A), left an enormous legacy of As contamination on and off these mine sites (e.g., INAC, 2009; Galloway et al., 2018; Van Den Berghe, Jamieson & Palmer, 2018; Palmer et al., 2019; Miller et al., 2019). Environmental concerns related to legacy As contamination spurred a number of remedial programs at several gold mine sites—for example, Giant Mine, Discovery Mine (1949–1969), Hidden Lake Mine (1959–1969), and Tundra Mine (1964–1968 and 1983–1986; INAC, 2009; Silke, 2009)—to restore these sites to a sustainable condition.

In 2002, the Crown Indigenous Relations and Northern Affairs Canada (CIRNAC) issued the Mine Site Reclamation Policy (MSRP) for the NT to enhance the environmental sustainability of mineral resources development (INAC, 2002). The policy stipulates that developers/owners of new and existing mine Projects must: (1) always have a closure and reclamation plan; (2) rehabilitate any onsite areas impacted by mining activities; and (3) monitor the progress of remedial efforts at rehabilitated sites post mine closure (INAC, 2002). While the impact of legacy As contamination has adversely affected a wide range of environments in the NT, contamination of lacustrine sediments and surface waters by As is of particular concern due to the socio-economic and ecological significance of lakes. Such concerns are mostly due to high toxicity and redox sensitivity of As, which allows it to vertically migrate within the sedimentary column post-deposition so that it may cycle between sediments and overlying waters (Toevs et al., 2006; Du Laing et al., 2009; Palmer et al., 2019). As a result of this mobility, lake sediments can either sequester As, typically under oxic conditions, or release As from sediment to porewaters, and subsequently to overlying surface waters under reducing conditions (Palmer et al., 2019). In some high latitude lakes that experience seasonal variability, including development of anoxia in winter under ice, this As cycling may be perpetual (Palmer et al., 2019). Monitoring programs focusing on lakes impacted by As contamination in the NT are often based on the collection and analyses of lake sediment and water samples (e.g., SENES Consultants Limited, 2011; Reid, 2012; AANDC, 2014; AECOM, 2015). An aspect that is usually overlooked by such programs is the biotic response to As contamination, especially that of benthic microfaunal communities (e.g., Nasser et al., 2016; Thienpont et al., 2016; Gavel et al., 2018; Sivarajah et al., 2019). Biological proxies can also provide information on the direct ecological impacts of contamination and have the advantage of not being post-depositionally re-mobilized (assuming a lack of bioturbation) as is the case for redox sensitive elements such as As.

Microbial benthic communities that preserve well in the sedimentary record have been shown to archive the impact of contamination on lacustrine ecosystems through time (e.g., Dixit, Dixit & Smol, 1989; Cattaneo et al., 2004; Gavel et al., 2018), and therefore have the potential to be a reliable tool for monitoring the efficacy of remediation efforts. Arcellinida, or testate lobose amoebae (Mitchell, Payne & Lamentowicz, 2008), are established bioindicators of change in lacustrine environmental conditions (Charman, 2001; Patterson & Kumar, 2002). These benthic protozoans are abundant and broadly distributed in Quaternary deposits in fresh and brackish aquatic environments, extending from the tropics to the Arctic (Medioli & Scott, 1988; Collins et al., 1990; Dalby et al., 2000; Charman, 2001; Patterson, 2014; Patterson et al., 2015). Arcellinida are characterized by tests (i.e., shells; size range = 15–200 μm) comprised of proteinaceous materials (autogenous tests) or assembled by agglutinating extraneous particles from the environment (xenogenous tests; Patterson & Kumar, 2002). Xenogenous tests have the greatest value in lacustrine studies owing to their high preservation potential.

The past 20 years have seen a growing interest in exploring the potential of Arcellinida as bioindicators of anthropogenic contamination (e.g., Asioli, Medioli & Patterson, 1996; Kumar & Patterson, 2000; Neville et al., 2011; 2014; Kihlman & Kauppila, 2012). Studies by Patterson, Baker & Burbridge (1996) and Reinhardt et al. (1998) were the first to confirm the sensitivity of Arcellinida to mine-derived As contamination at an intra-lake spatial scale in lakes from Ontario, Canada. Nasser et al. (2016) provided evidence of a strong quantitative relationship between Arcellinida assemblages and changes in sedimentary As concentrations in a regional spatial survey of near-surface sediments of 59 lakes in the Yellowknife area, NT. Recently, Gavel et al. (2018) demonstrated the sensitivity of Arcellinida assemblages to temporal increase in As concentrations associated with historic gold mining, in a freeze core from Frame Lake in the City of Yellowknife, NT. The sensitivity of Arcellinida to spatio-temporal changes in As levels coupled with their rapid reproduction and abundance in lake sediments makes the group particularly useful for environmental monitoring and assessments of remedial efforts.

As lakes are generally characterized by heterogenous substrates and multiple sub-environments (Last & Smol, 2001), development of an appropriate sampling strategy is essential to ensure accurate environmental monitoring of the progress of remedial efforts in impacted lakes using Arcellinida population dynamics. A temporal sampling approach (e.g., Gavel et al., 2018), while beneficial for long-term environmental monitoring, is often limited by the number of sedimentary cores collected—often collected from the *Z*_max_ of a basin. In contrast, intra-lake sampling often targets multiple sites within a lake to maximize spatial coverage and to ensure the identification of faunal gradients developed in response to local scale variability in As concentrations. An intra-lake sampling strategy is most suitable for monitoring the progress of remedial efforts within an impacted lake. However, the only two intra-lake investigations of arcellinidan response to As contamination (i.e., Patterson, Baker & Burbridge, 1996; Reinhardt et al., 1998), while successful in confirming the sensitivity of Arcellinida to As, involved a project design that only produced semi-quantitative results. Therefore, a quantitative assessment of the intra-lake response of Arcellinida distribution to sedimentary As concentration in near-surface sediment is still required to further develop the group as a reliable tool for monitoring As contamination in lakes.

In this study, we examine Arcellinida species and strains in 40 surface-sediment samples from two lakes in the central NT with the aim of quantifying their response to intra-lake variability of As concentrations and other controls. The studied lakes are Frame Lake (62.454°N, −114.390°E; *n* = 25; Fig. 1B), located within the City of Yellowknife, and Hambone Lake (64.046741 N, −111.156795 W; *n* = 15) located ~240 km northeast of the Yellowknife area (Fig. 1C). These lakes are known to have elevated levels of As derived at least in part from the respective operations of the former Giant (Frame Lake) and Tundra gold mines (Hambone Lake), and are currently the target of ongoing remedial and environmental monitoring efforts (e.g., Frame Lake rehabilitation project, led by Tides Canada; Tundra Mine remediation project, led by CIRNAC). The ecological health of Frame Lake, once a prominent recreational site in the City of Yellowknife, deteriorated markedly in only a decade from the late 1960s onward due to encroaching urbanization, aerial fallout of As from roaster stack emissions of Giant Mine, and suspected illegal dumping of sediments (Gavel et al., 2018; Menard et al., 2019). Elevated As levels in Hambone Lake are derived from the operations of the nearby Tundra Mine, and are the result of weathering of mine tailings and seepage from tailings containment ponds near the lakes western shore (Reid, 2012; Miller et al., 2019). As the former Giant and Tundra gold mines are known to have introduced different As mineral forms to surrounding lakes (Galloway et al., 2018; Schuh et al., 2018; Miller et al., 2019), a secondary objective of this study is to compare the impact of As derived from these mines on Arcellinida distribution.

### Regional setting

#### Frame Lake

The low-relief terrain surrounding the Yellowknife area consists of rocky outcrops associated with glacial and glaciolacustrine sediments in topographic lows (Stubley, 1997). The region has a continental subarctic climate with relatively cool, dry summers and cold and even dryer winters. Mean annual temperature is −4.3 °C with a mean annual precipitation of 288.6 mm (Environment Canada, 2019). Prevailing wind blows from the east for most of the year except for the months of June, July, and August, when it blows out of the south (SENES Consultants Limited, 2005; Environment Canada, 2019).

Frame Lake (62.454°N, −114.390°E) is a subarctic lake located near the downtown area of the City of Yellowknife (Fig. 1B). The lake is underlain by rocks of the Yellowknife Supergroup of the southern Slave structural province of the Canadian Shield in the central NT, Canada (Cousens, 2000). Bedrock lithologies consist of Archean meta-volcanic and meta-sedimentary rocks intruded by younger granitoids (Siddorn et al., 2007). Surficial sediments are primarily comprised of a thin (<2 m thick) discontinuous layer of till and Glacial Lake McConnell sediments (Kerr & Wilson, 2000). The till consists of a stony and loosely compacted matrix-supported diamicton (Smith, 1994; Kerr & Wilson, 2000) while the Lake McConnell sediments are comprised of poorly to moderately sorted coarse to fine sand, silt, and clay that can be up to 20 m thick in some topographic lows.

Frame Lake has a relatively small surface area (88.4 ha) with an elongate shape (southwest-northeast trend) that widens toward the northeastern end of the lake (Fig. 1B). The lake has a maximum length and width of 1.75 km and ~ 1 km, respectively (Healey & Woodall, 1973; Dirszowsky & Wilson, 2016), and a maximum depth of 4.5 m in the southern basin and 5.5 m in the northern basin (Menard et al., 2019). Vegetation around the lake includes conifers, paper birch and shrubs (Kerr & Wilson, 2000). Water derived from sheet wash or small ephemeral channels attributed to rainfall events and snowmelt are currently the primary source of inflow to Frame Lake (Dirszowsky & Wilson, 2016; Gavel et al., 2018). The lake’s outflow is currently controlled by the opening and closing of a sluice gate in a causeway, located on the lake’s eastern shore, which remains close most of the year (Gavel et al., 2018).

Before the 1970s, Frame Lake was an attractive recreational destination for residents and tourists alike (e.g., swimming at the McNiven bathing beach, and canoeing). The lake also used to serve as a fishing spot for the Yellowknives Dene, with both whitefish and pike being found (Tides Canada, 2015). However, historical mining activities in the area, especially that of Con (1938–1999) and Giant mines, coupled with the continuous urbanization of the City of Yellowknife, led to a severe decline in the ecological health of the lake (Dirszowsky & Wilson, 2016; Gavel et al., 2018; Menard et al., 2019). The lake is currently targeted for remedial and monitoring efforts (e.g., Frame Lake rehabilitation project; Tides Canada, 2015) to restore lake conditions to a state that is compatible with environmental guidelines and human activities.

#### Hambone Lake

Hambone Lake (64.046741 N, −111.156795 W) is located within the former Tundra Mine property in the Courageous Lake region, approximately 240 km northeast of the City of Yellowknife (Fig. 1C). Elevations rise gradually rise from 157 m above mean sea level near Frame Lake to 350–400 m above mean sea near Hambone Lake, over a distance = 240.44 km (Stubley, 1997). The region is characterized by a low-relief, gently rolling tundra that overlies bedrock belonging to the Yellowknife Supergroup in the central portion of the Slave structural Province (Dillion-Leitch, 1981; Hatfield Consulting, 1982; Gibson & Reid, 2014). The volcanic lithologies of the Courageous Lake Greenstone Belt lie unconformably on top of thin, discontinuous sequences of volcanic, clastic, and banded iron formations of the Central Slave Cover Group and the diorite and tonalite gneiss of the Central Slave Basement Complex, and are bordered by the Courageous Lake Batholith to the west and turbidite metasedimentary rocks to the east (Moore, 1951; Bleeker et al., 1999). Hambone Lake is situated within the Lockhart River drainage basin (catchment area ~ 26,600 km^2^), which drains southward into Great Slave Lake (Topographical Survey of Canada, 1925). The climate of the study region is cold and dry with an average annual temperature of −12 to −9 °C (SENES Consultants Limited, 2006).

Hambone Lake is relatively small with a surface area of only 10.7 ha (711.4 m long and 292.5 m wide). The name of the lake is derived from its unique morphology that resembles a hambone with a wider southwestern section that tapers towards the northeast (Fig. 1C). The lake is shallow, with an average depth of 1.5 m (maximum depth is ~ 2m; Gibson & Reid, 2014; Miller et al., 2019). Hambone Lake is well mixed in the open water season due to its shallow nature and the elevated frequency of high winds in the study area (Gibson & Reid, 2014). A drainage pathway from the Tundra Mine site, discharged from a tailings containment pond called Lower Pond passes directly through Hambone Lake and downstream through the lake’s outlet to Powder Mag Lake, Sandy Lake, and ultimately discharging into Courageous Lake (Reid, 2012; Gibson & Reid, 2014).

The Contaminants and Remediation Directorate of Crown Indigenous Relations and Northern Affairs Canada (CIRNAC (formerly AANDC and INAC) since 1999) is responsible for maintenance and care of the Tundra Mine site. The site has gone through various remediation activities that started in 2007 and ended recently in 2018 (e.g., INAC, 2005; SENES Consultants Limited, 2006, 2011; AANDC, 2014; AECOM, 2015, 2018). Currently, the mine site is part of long-term monitoring program, which began following the completion of all remedial efforts in 2018 (AECOM, 2018). Recently, Miller et al. (2019) has shown that levels of As in surface-sediment samples (top 0.5 cm) collected from Hambone Lake in 2016 remain well above the Canadian sediment quality guidelines for the protection of aquatic life (As concentration range = 195–622 mg kg^−1^; Interim Sediment Quality Level (ISQL) = 5.9 mg kg^−1^; Probable Effect Level (PEL) = 17 mg kg^−1^; Canadian Council of Ministers of the Environment (CCME), 2002).

## Materials and Methods

### Sampling design and field methods

Forty near-surface sediment samples were collected from Frame Lake (*n* = 25) and Hambone Lake (*n* = 15) during the summer field seasons of 2014 and 2016, respectively (Aurora Research Institute—Aurora College (Licence Nos. 14435, 14965 and 15852)). Lakes were accessed by a boat and surface-sediment samples were collected using an Ekman Grab sampler. The upper ~0.5 cm of sediment from each Ekman Grab was retained for Arcellinida, sedimentological and geochemical analysis using an inert plastic laboratory spoon. The coordinates for each sampled site within the lakes were recorded using a geographic positioning system (model: GPSMAP 76CSx; manufactured in Garmin Ltd., Olathe, KS, USA; Table S1). Water depth was determined for each sampling station using a HONDEX Honda portable handheld depth sounder (model: PS-7; manufactured in Aichi, Tokyo, Japan). Muddy substrates were preferentially selected for sampling, as nutrient poor silt to sand substrates are generally characterized by allochthonous arcellinidan populations (Patterson & Kumar, 2002).

### Laboratory methods

#### Particle size analysis

Particle size analysis (PSA) was performed on the sediment sub-samples to identify textural influence on the intra-lake distribution of Arcellinida and element concentrations at each station. Following the sample preparation protocols of Murray (2002) and Van Hengstum et al. (2007), PSA sub-samples were first digested in a heated bath (70 °C) with 10% HCl and 30% H_2_O_2_ to remove carbonate and organic content, respectively. Digested sub-samples were then anayzed for their sedimentary grain size using a Beckman Coulter LS13 320 laser diffraction analyzer (manufactured in California, USA) fitted with a universal liquid medium (ULM) sample chamber over a measurement range between 0.4 and 2,000 µm. Sub-samples were loaded into the instrument until an obscuration level of 10 ± 3% was attained. GRADISTAT (Version 8; Blott & Pye, 2001) was used to compile the results (Table S1). Garnet15 (mean diameter 15 μm: ± 2 μm), an accuracy standard supplied by Beckman Coulter, was run once per month, while an in-house mud sample (Cushendun Mud; mean diameter = 20.5 μm: ± 0.76 μm) was run at the start of every session as a precision control. Generated mean values for the analyzed Garnet15 (16.2 μm) and Cushendun (21.1 μm) fell within the standard deviation ranges for optimal instrument performance.

#### Geochemical analysis

Elemental concentrations of the sediment sub-samples were analyzed using ICP-MS using the *aqua regia* digestion protocol (ICP-MS 1F/AQ250 package) at Bureau Veritas, Vancouver (Table S1). *Aqua regia* digestion was employed instead of a 4-acid digestion protocol, as the former provides the total concentration of metal(loids) that could potentially become bioavailable, while the latter method can potentially volatilize non-bioavailable As (Parsons et al., 2012), which would generate results inappropriate for comparison with Arcellinida data. Analytical precision was assessed using duplicate samples. Calculated Relative Percent Difference (RPD) was less than 5% for As (RPD range = 0.196–1.379%, *n* = 3). Analytical accuracy was assessed using standard reference materials: (1) STD DS10 (*n* = 3); (2) STD D11 (*n* = 6); and (3) STD OREAS45EA (*n* = 9). Mean As concentration measured in STD DS10 was 44.8 mg kg^−1^ ± 1.63 (*n* = 3) compared to an expected concentration of 46.2 mg kg^−1^ (mean RPD = 3.69% ± 2.704). Mean As concentration measured in STD DS11 was 41.9 mg kg^−1^ ± 4.38 (*n* = 6) compared to an expected concentration of 42.8 mg kg^−1^ (mean RPD = 5.62% ± 3.908). Mean measured As concentration for STD OREAS45EA was 10.85 mg kg^−1^ ± 0.751 (*n* = 9) compared to an expected As concentration of 10.3 mg kg^−1^ (mean RPD = 7.23% ± 4.0065). Laboratory methods blanks (*n* = 11) resulted in As detection in two blanks (detected As concentrations = 0.2 mg kg^−1^ and 0.5 mg kg^−1^).

#### Olsen phosphorus (OP) extraction

Sediment-based phosphorus was measured to determine the trophic status of the lakes at Taiga Environmental Laboratory, Yellowknife. Phosphorus was examined using the Olsen phosphorus extraction method (Olsen-P; Olsen et al., 1954) as it provides a reliable measure of phosphorus that is bio-available to aquatic plants and macro- and micro-algal communities (Zhou, Gibson & Zhu, 2001).

#### Loss-on-ignition

Loss on Ignition (LOI) analysis was carried out on the 38 sub-samples (insufficient materials for samples FL1 and FL7) to determine the percentage of moisture, organic carbon, carbonate, and minerogenic content (Heiri, Lotter & Lemcke, 2001). Moisture content was determined by comparing measurements before and after samples were placed in an oven at 100 °C for 24 h. A Thermo Scientific Thermolyne Benchtop Muffle Furnace (model: F48025-60-80; manufactured in Massachusetts, USA) was then programmed for sequential burning at 550 °C and 900 °C to determine percentages of organic carbon and carbonate, respectively (Table S1). Analytical precision was assessed using five duplicate samples. Calculated RPD for moisture, organic carbon, carbonate, and minerogenic content was less than 7.5% (*n* = 38). Carbonate content was excluded from ensuing statistical analysis due to the high calculated RPD (range = 9.4–106.3%).

#### Micropaleontological analysis

Three cm^3^ of material was sub-sampled from each sample for micropaleontological analysis. These sub-samples were wet sieved through a 297 µm mesh to remove any coarse debris and then through a 37 µm mesh to separate Arcellinida from the fine particles (Patterson & Kumar, 2002). Sieved sub-samples were then subdivided into six aliquots for quantitative analysis using a wet-splitter (after Scott & Hermelin, 1993). Aliquots were analyzed wet for Arcellinida tests on a gridded petri dish using an Olympus SZH dissecting binocular microscope (7.5–64× magnification) until a statistically significant number of specimens were quantified (minimum of 150 tests; Patterson & Fishbein, 1989; Table S1). All sub-samples had statistically significant numbers of Arcellinida tests and were thus included in subsequent statistical analyses.

**Figure 2:**
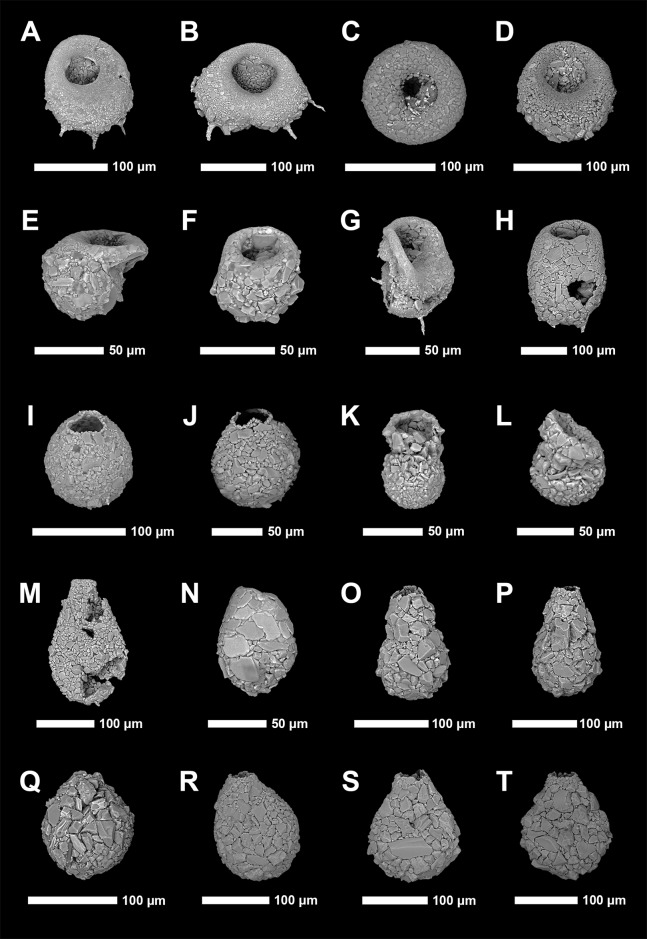
Scanning electron microscope plate of Arcellinida specimens from Frame Lake and Hambone Lake. (A and B) *Centropyxis aculeata* (Ehrenberg, 1832) stain “aculeata”. (C and D) *Centropyxis aculeata* (Ehrenberg, 1832) stain “discoides”. (E and F) *Centropyxis constricta* (Ehrenberg, 1843) stain “aerophila”. (G and H) *Centropyxis constricta* (Ehrenberg, 1843) stain “constricta”. (I and J) *Cucurbitella tricuspis* (Carter, 1856). (K and L) *Conicocassis pontigulasiformis* (Beyens, Chardez & De Bock, 1986) (Nasser & Patterson, 2015). (M) *Hyalosphenia papilio* (Leidy, 1874). (N) *Heleopera* sphagni (Leidy, 1874). (O and P) *Lagenodifflugia vas* (Leidy, 1874). (Q and R) *Lesquereusia spiralis* (Ehrenberg, 1840). (S and T) *Pontigulasia compressa* (Carter, 1864).

**Figure 3:**
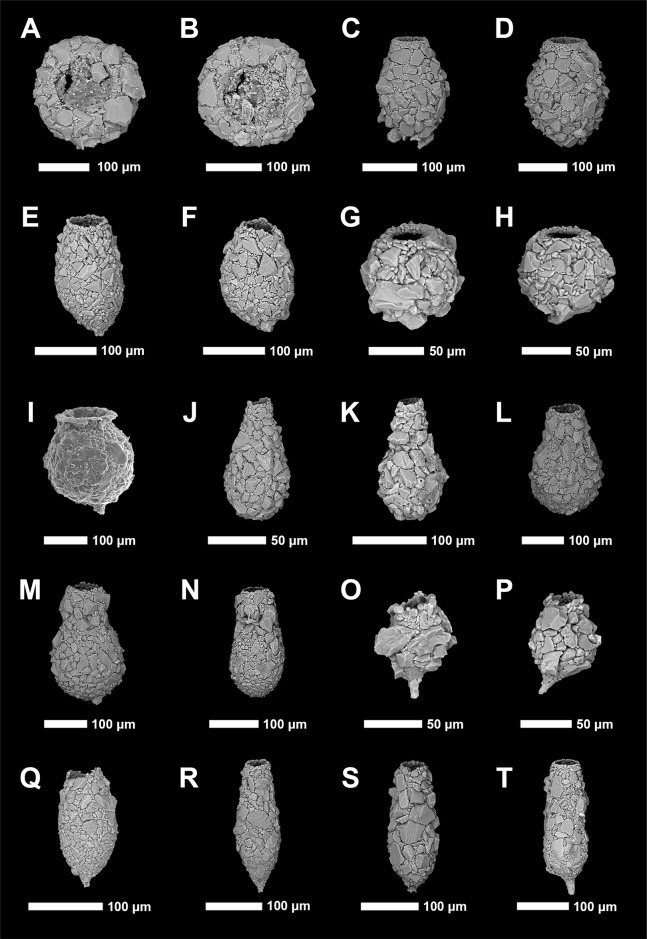
Scanning electron microscope plate of Arcellinida specimens from Frame Lake and Hambone Lake. (A and B) *Phryganella nidulus* Penard (1902). (C and D) *Difflugia glans* Penard (1902) strain “glans”. (E and F) *Difflugia glans* Penard (1902) strain “distenda”. (G and H) *Difflugia globulosa* (Dujardin, 1837). (I) *Difflugia urceolata* Carter (1864) strain “urceolata”. (J and K) *Difflugia oblonga* (Ehrenberg, 1832) strain “oblonga”. (L and M) *Difflugia oblonga* (Ehrenberg, 1832) strain “tenuis”. (N) *Difflugia oblonga* (Ehrenberg, 1832) strain “lanceolata”. (O and P) *Difflugia elegans* (Penard, 1890). (Q and R) *Difflugia protaeiformis* (Lamarck, 1816) strain “acuminata”. (S) *Difflugia protaeiformis* (Lamarck, 1816) strain “claviformis”. (T) *Difflugia curvicaulis* (Penard, 1899).

Identification of Arcellinida was primarily based on Reinhardt et al. (1998), Roe, Patterson & Swindles (2010), Patterson et al. (2013), Steele et al. (2018) and the extensive online Microworld database (Siemensma, 2019). Because lacustrine arcellinidan species can display considerable environmentally-controlled infraspecific morphological variability (e.g., Medioli & Scott, 1983), the accepted practice has been to designate informal infrasubspecific “strain” names for these ecophenotypes (Asioli, Medioli & Patterson, 1996; Reinhardt et al., 1998; Patterson & Kumar, 2002). While infrasubspecific level designations have no status under the International Zoological Code of Nomenclature (art. 45.5; 4th edition, 1999; International Commission on Zoological Nomenclature, 1999), they have been extensively used in the literature for defining environmentally significant populations within lacustrine environments (e.g., Reinhardt et al., 1998; Patterson & Kumar, 2000, 2002; Roe, Patterson & Swindles, 2010; Steele et al., 2018). Scanning electron microscope images of common species and strains were obtained using a Tescan Vega-II XMU VP scanning electron microscope (SEM) in the Carleton University Nano Imaging Facility. All SEM plates were digitally produced using Adobe Photoshop™ CC 2018 (Figs. 2 and 3).

### Statistical analyses

Twenty-five Arcellinida species and strains were identified in 40 near-surface sediment sub-samples (Table S1). The Probable Error (pe) was calculated for each sub-sample:

$${\rm pe} = 1.96\left( {\displaystyle{S \over {\sqrt {Xi} }}} \right)$$

where *S* is the standard deviation of the population count and *Xi* is the fractional abundance (Patterson & Fishbein, 1989). A sample count was deemed statistically insignificant if the probable error exceeded the total count for a sample. All samples contained statistically significant populations.

Standard error (*Sxi*) was calculated for each sample using the following equation:

$$Sxi = 1.96\sqrt {\displaystyle{{F1\left( {1 - F1} \right)} \over {Ni}}}$$

where *F*1 is the fractional abundance of species and *Ni* is the total number of counts. Species were considered to be present in insignificant number if the standard error exceeded the total counts for that species in all sub-samples (Patterson & Fishbein, 1989). All species and strains were found to be present in statistically significant numbers.

The Shannon Diversity Index (SDI; Shannon, 1948) was calculated using the vegan package in RStudio statistical software (version 0.98.1028; R Core Team, 2014) to assess arcellinidan diversity as a measure of ecological health in both lakes. Samples were considered healthy if the SDI was between 2.5 and 3.5, in transition if SDI was between 1.5 and 2.5, and stressed if SDI was below 1.5 (Magurran, 1988; Patterson & Kumar, 2002). The ratio between difflugid and centropyxid species (D/C; Neville et al., 2011) was used in tandem with SDI as a measure of the relative ecological health of the sampled lakes (Table S1).

#### Data screening, variables selection, and multivariate statistics

Any variables in the arcellinidan relative abundance, LOI, PSA, and ICP-MS data sets having issues associated with more than 25% of their values (i.e., missing values, below detection or above detection) were removed (Reimann et al., 2008). Geochemical determinations below the method detection limit were substituted with ½ of that limit. This screening procedure resulted in the removal of six variables from the ICP-MS data set (Te, Ge, Ta, In, Pd, and Pt; Table S1). A Principle Component Analysis (PCA; Pearson, 1901; Hotelling, 1933) plot was employed to evaluate redundancies in data and further reduce the number of parameters to be included in constrained ordination.

Q-mode and R-mode cluster analysis was carried out on the 25 arcellinidan species and strains in the 40 screened samples. Q-mode cluster analysis was employed to group samples according to similarities in their faunal composition using Ward’s Minimum variance method (Ward, 1963). R-mode cluster analysis was employed to determine which species and strains (*n* = 25) coexist with each other and identify the dominant taxa responsible for shaping the faunal structure of the identified assemblages. The statistical significance of the identified clusters was calculated using the PVClust package in RStudio. A two-way cluster analysis heatmap was generated using the heatmap.2 function of the gplots package in RStudio. Nonmetric Multidimensional Scaling (NMDS; Kruskal, 1964) was used to further investigate the results of cluster analysis by assessing the similarity between identified assemblages in multidimensional space.

Redundancy analysis (RDA; Van den Wollenberg, 1977) was used to evaluate the relationship between the identified arcellinidan assemblages and the measured parameters in both lakes. A series of partial RDAs (pRDA), coupled with variance partitioning tests, were also carried out to identify the significance of the variance in the arcellinidan distribution that is attributed to the analyzed measured variables. Variables with a *p* < 0.05 were considered to be significant contributors to variance in the arcellinidan assemblage. Data sets from Frame Lake (25 samples and 21 species and strains) and Hambone Lake (15 samples and 25 species and strains) were analyzed separately for RDA and pRDA due to the apparent hydroecological differences between both lakes.

## Results

### Intra-lake distribution of As

Median As concentrations in Frame Lake (270.8 mg kg^−1^; *n* = 25) and Hambone Lake (293.1 mg kg^−1^; *n* = 15) are relatively similar, but As concentrations are more variable in Frame Lake (As range = 145.1–1336.6 mg kg^−1^) compared to Hambone Lake (As range = 66.6–488.3 mg kg^−1^). Sedimentary As concentrations in all samples (median As concentration = 283.9 mg kg^−1^; range = 66.9–1336.6 mg kg^−1^; *n* = 40) are well above the Canadian sediment quality guidelines for the protection of aquatic life (ISQL = 5.9 mg kg^−1^; PEL = 17 mg kg^−1^; Canadian Council of Ministers of the Environment (CCME), 2002), and are notably higher than background As levels in Yellowknife area lake sediments (20–30 ppm; Galloway et al., 2015, 2018).

The highest As concentrations in Frame Lake were found in sites within and near the northern basin (median As concentration = 674.4 mg kg^−1^; range = 177.5–1336.6 mg kg^−1^; *n* = 5 (F17–F20)). The rest of the lake exhibited a patchy As distribution with no clear spatial trend (Fig. 1B). In Hambone Lake, the highest As concentrations were recorded in sites closer to the lake’s outlet at the lake’s northeastern margin (median As concentration = 386.6 mg kg^−1^; *n* = 4 (H1, H2, H3 and H4); Fig. 1C). Arsenic levels in the rest of the lake were slightly lower than those recorded in the vicinity of the outlet, but remain above the CCEM guidelines (median As concentration = 247.2 mg kg^−1^; *n* = 11).

### Description of identified arcellinida assemblages

**Figure 4:**
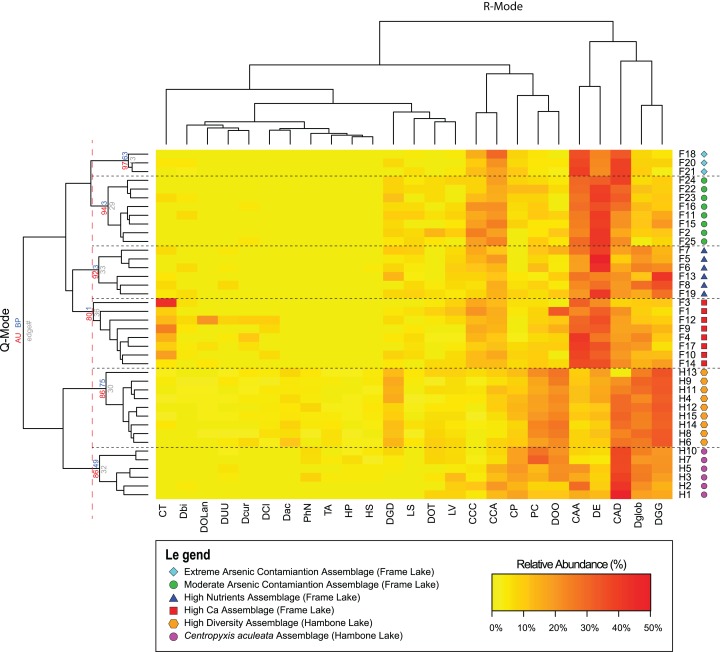
Arcellinida assemblages based on A two-way heatmap cluster analysis. The two-way heat map dendrogram shows the relative abundance of the 25 identified Arcellinida species and strains (R-mode; horizontal dendrogram) in the sample of the identified assemblages (Q-mode; vertical dendrogram). The coloured numbers on the Q-mode dendrogram represents the results of PVClust analysis that shows the statistical significance of the identified assemblages (red numbers).

Results of the Q-mode cluster analysis and PVClust allowed identification of six distinct arcellinidan assemblages, four in Frame Lake (assemblages 1–4) and two in Hambone Lake (assemblages 5 and 6): (1) extreme As contamination assemblage (EAC; approximately unbiased probability value (AU *p*-value) = 97%; *n* = 3); (2) moderate As contamination assemblage (MAC; AU *p*-value = 94%; *n* = 8); (3) high calcium assemblage (HC; AU *p*-value = 80%; *n* = 8); (4) high nutrients assemblage (HN; AU *p*-value = 92%; *n* = 6); (5) high diversity assemblage (HD; AU *p*-value = 86%; *n* = 9); and (6) *Centropyxis aculeata* assemblage (CAA; AU *p*-value = 86%; *n* = 6; Fig. 4). The R-mode cluster dendrogram revealed that the faunal composition of the identified assemblages is mostly impacted by five taxa: (1) *Centropyxis aculeata* (Ehrenberg, 1832) strain “aculeata”; (2) *Difflugia elegan*s (Penard, 1890); (3) *Centropyxis aculeata* (Ehrenberg, 1832) strain “discoides”; (4) *Difflugia globulosa* (Dujardin, 1837); and (5) *Difflugia glans* Penard (1902) strain “glans” (Fig. 4). Other species of a lesser, yet notable, influence includes *C. constricta* (Ehrenberg, 1843) strain “aerophila”, *C. constricta* (Ehrenberg, 1843) strain “constricta”, *Difflugia oblonga* (Ehrenberg, 1832) strain “oblonga” and *Cucurbitella tricuspis* (Carter, 1856) (Fig. 4).

#### Extreme As contamination assemblage

The extreme As contamination assemblage (EAC; *n* = 3) was identified in relatively deep (median water depth = 5.5 m; range: 3.5–6.4 m), organic-rich (median TOC % = 53.4%; range: 52.1–59.1%) and silt-dominated substrates (median Silt % = 79%; range: 78–83%) from the northern basin of Frame Lake (Fig. 1B). Arsenic concentrations in the EAC samples were the highest in Frame Lake (median As = 695.1 mg kg^−1^; range: 234.9–1336.6 mg kg^−1^).

The faunal density in EAC samples was also the highest in the lake (median = 600 tests per cm^3^; range = 110–760 tests per cm^3^) but the species richness was relatively low (median = 13 species; range: 11–14 species). The low to moderate diversity of the assemblage was reflected by the measured SDI values (range = 1.6–1.9) and is indicative of stressed to transitional environmental conditions (Magurran, 1988). The faunal assemblage structure of EAC was dominated by *C. aculeata* “discoides” (median relative abundance % = 33.9%; range = 25.6–34.7%) and *C. aculeata* “aculeata” (median relative abundance % = 32.1%; range = 29.3–35.5%). Elevated numbers of *D. elegans* (median relative abundance % = 11.5%; range = 11.3–13%) and *C. constricta* “constricta” (median relative abundance % = 10.3%; range = 7.1–21.3%) were also present in most samples. Indicators of healthy-environmental conditions, like *D. oblonga* “oblonga” (median relative abundance % = 0.26%; range = 0–0.6%), *D. globulosa* (median relative abundance % =1.82%; range = 1.3–2.3%); *C. tricuspis* (median relative abundance % = 0%; range = 0–0.6%), and *D. glans* “glans” (median relative abundance % = 0.67%; range = 0.26–1.82%), were present in low numbers.

**Figure 5:**
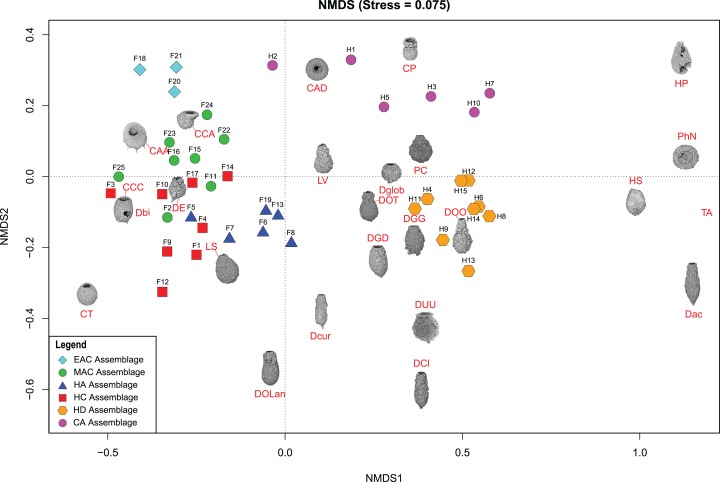
Nonmetric Multidimensional Scaling (NMDS) bi-plot. The coloured symbols represent the samples of the identified assemblages in Frame Lake and Hambone Lake. CCA, *Centropyxis aculeata* “aculeata”; CCD, *Centropyxis aculeata* “discoides”; CCA, *Centropyxis constricta* “aerophila”; CCC, *Centropyxis constricta* “constricta”; CT, *Cucurbitella tricuspis*; CP, *Conicocassis pontigulasiformis*; HP, *Hyalosphenia papilio*; HS, *Heleopera* sphagni; LV *Lagenodifflugia vas*; LS *Lesquereusia spiralis*; PC *Pontigulasia compressa*; PhN, *Phryganella nidulus*; DGG, *Difflugia glans* “glans”; DGD, *Difflugia glans* “distenda”; Dglob *Difflugia globulosa*; DUU, *Difflugia urceolata* “urceolata”; DOO, *Difflugia oblonga* “oblonga”; DOT, *Difflugia oblonga* “tenuis”; DOLan, *Difflugia oblonga* “lanceolata”; DE, *Difflugia elegans*; Dbi, *Difflugia bicornis*; DA, *Difflugia protaeiformis* “acuminata”; DCl, *Difflugia protaeiformis* “claviformis”; DCur, *Difflugia curvicaulis*.

**Figure 6:**
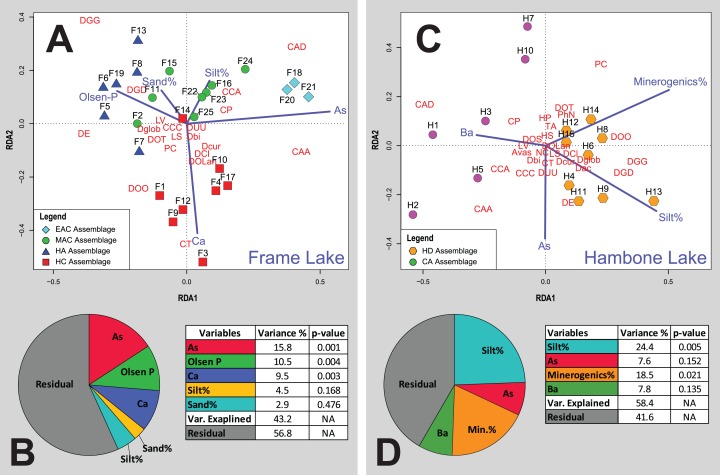
Redundancy analysis (RDA) tri-plots and variance partitioning results. (A) RDA triplot for the Frame Lake datasets. (B) Variance partitioning of the partial RDA of the Frame Lake dataset. (C) RDA triplot for the Hambone Lake datasets. (D) Variance partitioning of the partial RDA of the Hambone Lake dataset. The colored symbols represent the samples of the identified assemblages in Frame Lake and Hambone Lake. The dark blue lines represent the variables selected in both lakes. CCA, *Centropyxis aculeata* “aculeata”; CCD, *Centropyxis aculeata* “discoides”; CCA, *Centropyxis constricta* “aerophila”; CCC, *Centropyxis constricta* “constricta”; CT, *Cucurbitella tricuspis*; CP, *Conicocassis pontigulasiformis*; HP, *Hyalosphenia papilio*; HS, *Heleopera* sphagni; LV *Lagenodifflugia vas*; LS *Lesquereusia spiralis*; PC *Pontigulasia compressa*; PhN, *Phryganella nidulus*; DGG, *Difflugia glans* “glans”; DGD, *Difflugia glans* “distenda”; Dglob *Difflugia globulosa*; DUU, *Difflugia urceolata* “urceolata”; DOO, *Difflugia oblonga* “oblonga”; DOT, *Difflugia oblonga* “tenuis”; DOLan, *Difflugia oblonga* “lanceolata”; DE, *Difflugia elegans*; Dbi, *Difflugia bicornis*; DA, *Difflugia protaeiformis* “acuminata”; DCl, *Difflugia protaeiformis* “claviformis”; DCur, *Difflugia curvicaulis*.

Cluster analysis and NMDS showed that EAC samples clustered closely together (Figs. 4 and 5). The EAC samples varied positively with As and, to a lesser extent, silt particle size fraction and negatively with Olsen-P and sand particle size fraction on the RDA tri-plot (Fig. 6A).

#### High calcium assemblage

Samples hosting the High Calcium Assemblage (HC; *n* = 8) were collected from the southern section of Frame Lake except for samples F14 and F17, which were from the northern end (Fig. 1B). Th HC assemblage was found in relatively shallow to moderately deep substrates (median water depth = 2 m; range: 1.1–3.2 m) dominated by the silt particle size fraction (median Silt % = 79%; range: 15–82%) and characterized by relatively high organic content (median TOC % = 36%; range: 20–52.8%). Arsenic concentrations in HC samples are high (median = 302.1 mg kg^−1^; range = 151.1–492.3 mg kg^−1^) but are notably lower than the concentrations in EAC-bearing samples.

Samples of HC are characterized by notably lower faunal density compared to the EAC samples (median = 287 tests per cm^3^; range = 123–976 tests per cm^3^). An exception is sample F4, which is characterized by the highest faunal diversity in Frame Lake (976 tests per cm^3^). Species richness of HC is the highest in Frame Lake with a median of 17 species (range: 13–20 species). The measured SDI values for the samples of the assemblage (1.8–2.3) is reflective of environmental conditions transitioning toward more hospitable conditions (Magurran, 1988). Similar to EAC, the faunal structure of the assemblage is dominated by *C. aculeata* “aculeata”, albeit present in lower numbers (median relative abundance % = 25.9%; range = 12–39.9%). The notable increase in the numbers of *D. elegans* in HC (median relative abundance % = 23.5%; range = 11–28.5%) resulted in the taxa co-dominating the assemblage along with *C. aculeata* “aculeata”. The numbers of *C. aculeata* “discoides” (median relative abundance % = 8.3%; range = 1.6–11.1%) and *C. constricta* “aerophila” (median relative abundance % = 4.19%; range = 2.8-6%) declined in the samples of HC. In contrast, the proportions of *D. oblonga* “oblonga” (median relative abundance % = 2%; range = 0–25.1%), *D. globulosa* (median relative abundance % =4.4%; range = 2.1–12.3%), *D. glans* “glans” (median relative abundance % = 2.7%; range = 0.7–8.7%), and especially *C. tricuspis* (median relative abundance % = 4.4%; range = 0.6–43%) increased notably in HC.

Cluster analysis and NMDS showed that samples of the HC cluster closely together, with only F3 and F14 plotting more distinctly from the assemblage on the NMDS bi-plot (Figs. 4 and 5). The results of RDA reveal a strong positive association between the HC samples and Ca and a negative association with the particle size analysis parameters, sand and silt fractions (Fig. 6A).

#### Moderate As contamination assemblage

The Moderate As Contamination Assemblage (MAC; *n* = 8) inhabits relatively shallow (median water depth = 1.05 m; range: 0.7–1.5 m), organic-rich (median TOC % = 55.7%; range: 42.3–59.4%) and are silt-dominated (median Silt % = 77%; range: 72–81%) substrates in the northern section of Frame Lake, with two outliers from the southern section of the lake (samples F2 and F11; Fig. 1B). Arsenic concentrations in MAC samples (median = 240.8 mg kg^−1^; range = 145.1–295.3 mg kg^−1^) are slightly lower than the levels characterizing the HC samples.

The faunal density of samples hosting the MAC is comparatively higher than that of the HC (median = 364 tests per cm^3^; range = 81–367 tests per cm^3^). Species richness of MAC is low and is relatively similar to that of EAC (median = 13 species; range: 12–16 species). The measured SDI values for the samples of the assemblage (1.7–2.1) indicate transitional environmental conditions (Magurran, 1988). Unlike the HC and EAC, the faunal structure of MAC is dominated by *D. elegans* (median relative abundance % = 36.1%; range = 22.9–40.1%). Abundance of *C. aculeata* “aculeata” (median relative abundance % = 17.9%; range = 12.5–20.6%) remain high but is lower than in EAC and HC. Abundances of other centropyxiid taxa like *C. aculeata* “discoides” (median relative abundance % = 14.6%; range = 6.5–34.6%), *C. constricta* “aerophila” (median relative abundance % = 8.8%; range = 5.3–14.2%), and *C. constricta* “constricta” (median relative abundance % = 7%; range = 1.6–14.3%) are high compared to HC and EAC samples. Stress-sensitive taxa like *D. oblonga* “oblonga” (median relative abundance % = 0.6%; range = 0–2.9%), *D. globulosa* (median relative abundance % =2.7%; range = 0–3.6%), *D. glans* “glans” (median relative abundance % = 2.6%; range: 1.3–2.9%), and *C. tricuspis* (median relative abundance % = 0%; range = 0–1.1%) were present in low numbers.

The MAC samples group together on the cluster analysis dendrogram and NMDS bi-plot (Figs. 4 and 5). MAC and HC samples show considerable overlap on the NMDS bi-plot (Fig. 5). The RDA plot shows a positive association between the MAC and Olsen P, sand particle size fraction, and silt particle size fraction and a negative association with Ca (Fig. 6A).

#### High nutrients assemblage

Samples composing the High Nutrients Assemblage (HN; *n* = 6) are mostly found on substrates in the southwestern section of the lake except for samples F2 and F11, which are from the northern section of the lake (Fig. 1B). Samples were collected from relatively shallow water substrates (median water depth = 1.1 m; range: 01–4.6 m) that are characterized by high organic content (median TOC % = 43.5%; range: 16.8–61.3%) and silty sediments (median Silt % = 78%; range: 70–86%). Arsenic concentrations characterizing the HN samples are the lowest in Frame Lake with a median of 216.4 mg kg^−1^ (range = 168.8–346 mg kg^−1^).

The faunal abundance in the HN assemblage was the second highest in Frame Lake (median = 488 tests per cm^3^; range = 380–634 tests per cm^3^). The assemblage exhibited a moderate species richness with a median of 15 species (range = 12–7 species). The SDI values for the HN samples (1.5–2) are reflective of stressed to transitional environmental conditions (Magurran, 1988). However, sample F5 was the only sample with a relatively low SDI value in HN (1.5). Therefore, the assemblage is more indicative of transitional conditions when F5 is excluded (1.8–2).

The assemblage was dominated by *D. elegans* (median relative abundance % = 37%; range = 12.9–57.3%), with lower numbers of *C. aculeata* “aculeata” (median relative abundance % = 10.4%; range = 7.6–17%), *C. aculeata* “discoides” (median relative abundance % = 8%; range = 2.2–10.4%), *C. constricta* “aerophila” (median relative abundance % = 4.4%; range = 3.1–8.8%), and *C. constricta* constricta (median relative abundance % = 2.2%; range = 0.7–6.3%). Some stress-sensitive taxa like *D. oblonga* “oblonga” (median relative abundance % = 1.3%; range = 0.3–4.7%) and *C. tricuspis* (median relative abundance % = 0.56%; range = 0–1.1%) showed a minor increase in abundance compared to other healthy-conditions indicators like *D. globulosa* (median relative abundance % =10.7%; range = 3.1–18.6%), *D. glans* “glans” (median relative abundance % = 14.5%; range: 4.2–41.6%) that increased drastically in the samples of HN.

Results of the cluster analysis and NMDS revealed a close association between the HN samples (Figs. 4 and 5). The RDA tri-plot showed a positive association between the HN and Olsen-P and a negative association with As (Fig. 6A).

#### High diversity assemblage

The high diversity assemblage (HD; *n* = 9) occurred in samples collected from relatively shallow (median water depth = 1.5 m; range: 0.9–1.9 m), organic-rich (median TOC % = 26.6%; range: 12.7–31.9%), silt-dominated (median Silt % = 87.5%; range: 85.7–89.9%) substrates in and around the western basin of Hambone Lake (Fig. 1C). The median As concentration was 293.1 mg kg^−1^ (range = 91.7–488.3 mg kg^−1^).

The assemblage was characterized by the highest faunal density (median = 1386 tests per cm^3^; range = 956–2632 tests per cm^3^) and species richness (median = 20 species; range: 20–26 species) in both lakes. The measured SDI values for the samples of the assemblage (2.1–2.3) are reflective of environments transitioning to more hospitable conditions (Magurran, 1988). The faunal structure of the assemblage was dominated by *D. glans* “glans” (median relative abundance % = 24.9%; range: 22.2–27.5%). The assemblage was also characterized by elevated numbers of *C. aculeata* “discoides” (median relative abundance % = 16.5%; range = 0–21.8%), *D. globulosa* (median relative abundance % =15.1%; range = 11.4–21.6%), *D. oblonga* “oblonga” (median relative abundance % = 12.9%; range = 9.9–17.6%), and *Pontigulasia compressa* (Carter, 1864) (median relative abundance % = 7.7%; range = 5.8–12.8%). Other species like *D. glans* Penard (1902) strain “distenda” (median relative abundance % = 3.7%; range = 1.7–6.2%), *C. aculeata* “aculeata” (median relative abundance % = 2.3%; range = 0.9–3.2%), *Conicocassis pontigulasiformis* (Beyens, Chardez & De Bock, 1986; Nasser & Patterson, 2015) (median relative abundance % = 2.5%; range = 1.4–4.6%), and *C. constricta* “aerophila” (median relative abundance % = 1.7%; range = 0.4–3.4%) are present in low proportions.

Cluster analysis and NMDS results show the samples of HD clustering closely and distinctly from other Frame Lake assemblages (Figs. 4 and 5). The results of RDA revealed a positive association between the HD samples and loss-on-ignition-determined minerogenic content and the silt particle size fraction and a negative association with barium (Ba; Fig. 6C).

#### Centropyxis aculeata assemblage

Samples hosting the *Centropyxis aculeata* Assemblage (CA; *n* = 6) occurred in relatively shallow (median water depth = 1 m; range: 0.9–1.7 m), organic-rich (median TOC% = 37.7%; range: 11.3–52.1%), silt-dominated (median Silt % = 85.2%; range: 83–87.5%) substrates from the southern part of the western basin (H10) and eastern arm of Hambone Lake (H1, H2, H3, H5, H7; Fig. 1B). Arsenic concentrations (median = 277.5 mg kg^−1^; range = 66.9–422.8 mg kg^−1^) were slightly lower than those of HD.

The faunal density of the assemblage was lower than that of HD (median = 583 tests per cm^3^; range = 458–2,936 tests per cm^3^) except for two samples with exceptionally high faunal density (H7 = 2,936 tests per cm^3^ and H10 = 1,150 test per cm^3^). The species richness of the CA was notably lower than that of HD with a median of 16 species (range of 12–17 species). SDI values for the assemblage (1.7–2.1) are reflective of transitional environmental conditions (Magurran, 1988). Unlike the HD, the faunal structure of the CA assemblage was dominated by *C. aculeata* “discoides” (median relative abundance % = 32.7%; range = 29.3–55.5%). Elevated numbers of *D. globulosa* (median relative abundance % = 12.4%; range = 7.4–15.4%) and *D. oblonga* “oblonga” (median relative abundance % = 10.5%; range = 1.6–11.9%) were present but were slightly lower than the numbers characterizing the HD samples. Other species like *D. glans* “glans” (median relative abundance % = 8.9%; range = 4.7–9.8%), *C. pontigulasiformis* (median relative abundance % = 7.3%; range = 4.3–9.3%), and *C. aculeata* “aculeata” (median relative abundance % = 5.7%; range = 0.7–19.4%) were present in notable numbers. Species and strains like *C. constricta* “aerophila” (median relative abundance % = 3.7%; range = 1–3.2%), *Pontigulasia compressa* (median relative abundance % = 4.5%; range = 1.6–22.8%) were present in low numbers, with the exception of notably high numbers of *P. compressa* in samples H7 and H10.

The results of the cluster analysis and NMDS show that the samples characterizing the CA cluster closely (Figs. 4 and 5). The results of RDA revealed a positive association between the CA samples and Ba and a negative association with the LOI-determined minerogenic content and silt particle size fraction (Fig. 6C).

## Discussion

### Controls over the intra-lake arcellinida distribution

Cluster analysis led to identification of distinct arcellinidan assemblages from Frame Lake (*n* = 4) and Hambone Lake (*n* = 2). The unique faunal structure of these assemblages coupled with the lack of overlap between samples from each lake in the statistical results is reflective of varying intralake hydroecological conditions. The RDA and variance partitioning results support this assessment by showing different sets of variables driving the arcellinidan distribution within both Frame Lake and Hambone Lake (Fig. 6). The statistical results suggest that As may be the dominant overarching driver of assemblage structure in Frame Lake, while having a more subtle impact on the arcellinidan populations in Hambone Lake (Fig. 6). Nonetheless, our results confirm the impact of As on the intra-lake arcellinidan distribution in both lakes, albeit at different magnitudes.

#### Frame Lake

Of the controls on arcellinidan distribution identified in Frame Lake, Ca (9.5%; *p*-value < 0.004), Olsen-P (10.5%; *p*-value < 0.005), and especially As (15.8%; *p*-value < 0.003) contributed the most toward explaining the variance in the arcellinidan distribution. The observation that As is the most significant control in Frame Lake is not surprising given the results of previous studies that have identified the impact of As contamination on the lake’s ecology and sediment quality (Dirszowsky & Wilson, 2016; Gavel et al., 2018; Menard et al., 2019). The SDI values for the identified assemblages (SDI range = 1.5–2.3; *n* = 25) are typical of transitional lacustrine environments (Magurran, 1988; Patterson & Kumar, 2002). However, samples with elevated As concentrations, such as the EAC from the north basin, where As concentrations are the highest in Frame Lake, were associated with lower faunal diversity and higher proportions of stress-tolerant Arcellinida taxa. The faunal structure of the low diversity EAC is dominated by *Centropyxis* species and strains (D/C range = 0.18–0.24), which is expected given the ability of the opportunistic centropyxids to withstand varying level of environmental stress including that caused by As contamination (Patterson, Baker & Burbridge, 1996; Reinhardt et al., 1998; Nasser et al., 2016; Gavel et al., 2018). Similar trends are observed in the samples of HC and MAC. However, a notable reduction in As concentrations in these samples, compared to levels observed in the EAC samples, is associated with a major decline in the number of stress-tolerant taxa and a concurrent increase in faunal diversity and number of more sensitive fauna (e.g., difflugiids; D/C range = 0.4–1.6). The increase in the abundance of healthy conditions-indicating species and strains is most evident in the samples of HN (D/C range = 1.7–3.4) and is partly associated with the notable reduction in As concentration at these stations, which are the lowest across the lake.

The faunal response to As contamination in assemblages from the southern part of Frame Lake appears to be obscured by the influence of variables pertaining to nutrient loading (i.e., Olsen-P, Ca; total faunal variance explained, 10.5% and 9.5%, respectively). This assessment is confirmed by the RDA results that show HC and HN correlating strongly with Ca and Olsen-P, and weakly with As. Such results suggest that the faunal structure of these assemblages was likely shaped via the combined influence of these three environmental controls rather than As alone. Concentrations of Olsen-P are generally low in Frame Lake (median Olsen-P concentrations = of 44 mg kg^−1^; *n* = 25) and are highest in samples from the southern part of the lake (median Olsen-P range = 49.5–60 mg kg^−1^; *n* = 14; Table S1). Elevated levels of Olsen-P in and around the southern basin may be attributed to inputs from nearby residential and private facilities as well as municipal storm water drainage outflow (Gavel et al., 2018; Menard et al., 2019). Olsen-P is a good indicator of bioavailable phosphorus in soils and lakes, with higher concentrations indicating nutrient rich, productive, and eutrophic conditions (Zhou, Gibson & Zhu, 2001). A few studies have demonstrated the significance of Olsen-P on the distribution of Arcellinida and the association of higher numbers of healthy conditions-indicators (e.g., difflugiids and *C. tricuspis*) with elevated Olsen-P concentrations (Roe, Patterson & Swindles, 2010; Patterson, Roe & Swindles, 2012). Interestingly, the highest Olsen-P levels in our study were associated with a drastic increase in the numbers of *D. elegans* in HC and HN (Table S1). Elevated proportions of *D. elegans* has previously been linked to substrates contaminated by As in the Yellowknife area (Nasser et al., 2016) and elsewhere (Reinhardt et al., 1998; identified as *Difflugia protaeiformis* strain “amphoralis”). However, the increase in the proportions of *D. elegans* in this study was associated with a trend of declining As concentrations (Table S1). Patterson & Kumar (2000) have also linked the abundance of *D. elegans* (also identified as *D. protaeiformis* “amphoralis”) with the availability of their preferred food source, pennate diatoms. The abundance and distribution of diatoms has been shown to be impacted by the availability of phosphorus, which is a limiting nutrient that controls the productivity of plants as well as macro-and micro-algal communities in freshwater systems (e.g., Boström, Persson & Broberg, 1988; Anneville, Gammeter & Straile, 2005; Berthon et al., 2014; Brembu et al., 2017). While diatoms were not identified and quantified in this study, the increase in *D. elegans* numbers may reflect a similar increase in the proportions of pennate diatoms, which may in turn be attributed to the relatively higher bioavailability of P (i.e., higher Olsen-P) concentrations in the southern part of Frame Lake.

As observed with the Olsen-P concentrations, the highest measured Ca values are exclusive to the southern part of Frame Lake, specifically in the samples hosting HC (Table S1). The RDA plot reveals a close association between HC samples, Ca, and *C. tricuspis* along the second RDA axis (Fig. 6). This association is expected as samples of HC are characterized by the highest concentrations of Ca along with the highest numbers of *C. tricuspis*. The abundance of *C. tricuspis* has been linked with organic rich substrates and eutrophic conditions (Collins et al., 1990; Patterson et al., 2013). Several studies have suggested a link between elevated Ca concentrations and enhanced lake productivity (Otsuki & Wetzel, 1974; Kelts & Ksü, 1978; Behbehani et al., 1986). Therefore, the increase in *C. tricuspis* coupled and with a general increase in the faunal diversity and numbers of healthy lake-indicating taxa in HC may be indicative of enhanced lake productivity in the southern part of the lake.

#### Hambone Lake

The silt particle size fraction (variance explained = 24.4%; *p*-value = 0.005) and minerogenic content (variance explained = 18.5%; *p*-value = 0.021) were identified as the most significant drivers of arcellinidan distribution in Hambone Lake (Fig. 6D). Arcellinida populations and their distribution have been shown to be sensitive to changes in the sedimentary composition of lacustrine substrates, with silt- to mud-dominated substrates often hosting faunally diverse and dense arcellinidan assemblages, while sand-dominated substrates are characterized by allochthonous and stress-indicating assemblages (Roe & Patterson, 2006; Steele et al., 2018). The sedimentary composition of the Hambone Lake assemblages differed in that CA samples have slightly higher sand concentrations and notably lower minerogenic content (median sand% = 7.3%; median minerogenic% = 61%; *n* = 6) compared to the HD samples (median sand% = 3.1%; median minerogenic% = 72.4%; *n* = 9; Table S1). These differences are supported by the RDA plot that reveal a close correlation between the samples of HD, silt particle size fraction, and minerogenic content, and a negative association between these variables and CA samples along the first RDA axis (Fig. 6C). While such results may provide an explanation for the relatively higher faunal diversity in the HD samples, it is difficult to fully attribute the relative decline in Arcellinida diversity and species richness and density in CA samples to the meager increase in the sand particle size fraction, or loss of minerogenic content, especially when the sedimentary composition of samples hosting both assemblages are silt-dominated (HD median silt % = 87.5%; *n* = 9; CA median silt% = 85.2%; *n* = 6). Such decline in arcellinidan diversity and abundance may be attributed to the impact of As contamination.

The RDA results show As plotting along the second RDA axis, indicating a lower influence of As over the distribution of Arcellinida population in Hambone Lake (Fig. 6C). These results are surprising given the well documented impact of As contamination on the lake’s sediment and water quality (e.g., INAC, 2005; SENES Consultants Limited, 2006; Reid, 2012; Gibson & Reid, 2014; Miller et al., 2019). However, the measured As concentrations were higher in sites close to the lake’s outlet in the northeastern end (median As concentration = 386.6 mg kg^−1^; *n* = 4 (H1, H2, H3 and H4); Fig. 1C). Miller et al. (2019) have identified even higher As concentrations in two sites near the outlet and away from the treated tailings effluents discharge location on the western end (HAM-1 As concentration = 622 mg kg^−1^; HAM-2 As concentration = 576 mg kg^−1^). The study has also identified authigenic sulfides as the primary As solid phase in the lake, and interpreted its predominance as a sign of a continuous downstream post-deposition mobilization of As. Interestingly, the identified arcellinidan assemblages exhibited a subtle response to this downstream migration of As. Samples of CA near the lake’s outlet were associated with notably higher abundances of stress-indicating Arcellinida taxa, lower numbers of stress-sensitive taxa (D/C range = 0.2–1.1), lower faunal diversity (SDI range = 1.6–2.1), and lower species richness (median species richness = 16 species; range = 12–17 species) and species density (median species density = 583 tests per cm^3^) compared to samples of HD (Table S1). These results suggest that As, while identified as a weak control in Hambone, may still be impacting the intra-lake Arcellinida distribution in the lake.

### Consistency of the arcellinidan response to As contamination

A secondary objective of this study was to determine whether exposure to As derived from the Giant and Tundra mines, but in different minerogenic form, would trigger a similar or different arcellinidan response in Frame Lake and Hambone Lake. The limited, yet growing, body of research on the effects of As contamination on the spatio-temporal distribution of Arcellinida has consistently recognized a significant faunal shift from healthy to stressed assemblages in response to increasing As concentrations (Patterson, Baker & Burbridge, 1996; Reinhardt et al., 1998; Nasser et al., 2016; Gavel et al., 2018). While the same faunal shift in response to As was observed in both the Frame Lake and Hambone Lake assemblages, it was pronounced in the former and more subtle in the latter. This disparity in the faunal response to As is in line with the RDA and variance partitioning results, which indicate a strong As control over the intra-lake distribution of Arcellinida in Frame Lake and a much weaker influence in Hambone Lake (Fig. 6). With the exception of a few very high As measurements in Frame Lake’s northern basin, As concentration in both Lakes were similar (Frame Lake median As = 270.8 mg kg^−1^; *n* = 25; Hambone Lake median As = 293.1 mg kg^−1^; *n* = 15). Such similarity, therefor, eliminates variance in As concentration as an explanation to this difference in the influence of As control over the arcellinidan distribution in each lake. A likely explanation, given the history of Giant and Tundra mines, is that Arcellinida species and strains in each lake may be responding to As that is derived from a different mineral source.

The mining practices at the former Giant and Tundra mines introduced different forms of As mineralization to area lakes and other environments in their surrounding (Galloway et al., 2018; Schuh et al., 2018; Miller et al., 2019). The use of roasting at the Giant Mine to liberate gold from the hosting arsenopyrite led to the release of massive amounts of As_2_O_3_ to the atmosphere (20,000 tonnes before 1963; Wrye, 2008), which eventually accumulated in the sediments of many lakes around the mine (Galloway et al., 2018, Schuh et al., 2018). Gold at the Tundra Mine was associated with As-bearing sulfides, mainly arsenopyrite from hydrothermal mineralization, and was predominantly separated using the free-milling process (Ransom & Robb, 1986). Disposal and weathering of mine tailings, seepage from nearby tailings containment ponds, and discharge of treated tailings effluent have been identified as the primary pathways of As into lakes around the Tundra Mine site (Reid, 2012; Miller et al., 2019). Compared to naturally occurring As sulfides (e.g., arsenopyrite), roaster-derived As_2_O_3_ is more soluble, bioavailable, and bioaccessible (Plumlee & Morman, 2011). The dissolution of sediment bound As_2_O_3_ often results in the release of toxic species of As (As^3+^ and As^5+^) into sediment porewater. When porewater conditions are reductive, these As species will then diffuse toward the overlaying surface water and as a result of hydrologic redox conditions in the lake bottom sediments often increase in concentrations near the sediment-water interface, thus elevating the risk of As exposure for arcellinidan communities living there. While As_2_O_3_ is yet to be identified as the dominant As host in Frame Lake, due to the complicated protocols required to identify As species in lake sediments (see Galloway et al., 2018), it is reasonable to assume that the elevated As concentrations in the lake are of an anthropogenic origin and are particularly derived from the diagenesis of As_2_O_3_. This assessment is supported by the results of studies in the Yellowknife area, NT that have demonstrated the persistence of As_2_O_3_ in lake sediments (Galloway et al., 2018; Schuh et al., 2018). Additionally, Van Der Berge et al. (2017) have recently identified soluble As_2_O_3_ particles to be the primary source of As in sediment porewater in three lakes near the City of Yellowknife. Therefore, the pronounced response of the Frame Lake Arcellinida assemblages, along with statistical significance of As control, may be attributed to prolonged arcellinidan exposure to As contamination due to the continuous supply of As from dissolving solid phase As_2_O_3_. In contrast, the weaker arcellinidan response to As and the insignificant As control in Hambone Lake is likely more reflective of the dominance of a different As mineral form in the lake sediments. Miller et al. (2019) have identified authigenic As-bearing sulfides as the primary host of As in near-surface sediments of Hambone Lake. The low solubility of As sulfides, compared to As_2_O_3_, renders them less bioavailable and bioaccessible (Plumlee & Morman, 2011). This reduced As bioavailability may have resulted in a less frequent release of As into porewaters and concomitant, weaker faunal response in Hambone Lake.

Research focusing on the nature and type of the dominant As species and mineral forms in impacted lakes in the central NT is ongoing. However, the progress of this line of research is currently slowed down by the elevated cost of sample analysis and complicated nature of speciation protocols. Because of these limitations, As speciation analysis is only undertaken once preliminary geochemical analysis (e.g., ICP-MS) is conducted to evaluate the level of As contamination and the possibility of post-depositional mobility of As. The results of this study provide new insight into the value of using Arcellinida not only for monitoring As contamination but also the potential of using the group as reconnaissance tool to guide further research into As speciation by identifying impacted lakes where As is likely to be mobile and bioavailable due to the nature of the source As mineralization.

## Conclusions

This research quantified the intra-lake relationship between Arcellinida fauna and variability of As derived from two former gold mines, and other lake environmental variables through analysis of 40 sediment-water interface samples from Frame Lake (*n* = 25) in the Yellowknife area, and Hambone Lake in the Courageous Lake region (*n* = 15).

Interpretation of statistical and multivariate analyses led to the identification of distinct arcellinidan assemblages in Frame Lake (*n* = 4) and Hambone Lake (*n* = 2) that reflect hydroecological conditions unique to each lake. Arsenic was found to be the most statistically significant control over the distribution of Arcellinida assemblages identified in Frame Lake while having a marginal effect on the assemblages found in Hambone Lake. We attribute such disparity in the significance of relative As influence on the arcellinidan ecology in each lake to the variance in the dominant As mineralization in Frame Lake (As_2_O_3_) and Hambone Lake (As-sulphides), which is in turn related to the documented difference in ore-processing practices at the former Giant (roasting) and Tundra mines (free-milling).

Results from this study provide quantitative evidence for the sensitivity of Arcellinida to varying levels of As contamination derived from different As mineral forms and shows the potential of using this group as a reliable tool for monitoring As contamination, lacustrine ecological health, and the progress of remedial efforts in impacted lakes. These results are also significant as they showcase the potential of using Arcellinida as a reconnaissance tool to discriminate the dominant As mineral forms, which in turn will further guide the selection of impacted lakes for As speciation analysis.

## Supplemental Information

10.7717/peerj.9054/supp-1 Supplemental Information 1 Lake information. Loss-on-ignition, Olsen-P, particle size analysis (PSA), ICP-MS results, and Arcellinida relative abundance results in lake surface-sediment samples (*n* = 40) from Frame Lake and Hambone Lake in the central Northwest Territories, Canada.

## Additional Information and Declarations

### Field Study Permissions

The following information was supplied relating to field study approvals (i.e., approving body and any reference numbers): Field experiments were approved by the Aurora Research Institute—Aurora College (Licence Nos. 14435 and 14965 for the Yellowknife area portion and Licence No. 15852 for the Courageous Lake area portion).

### Data Availability

The following information was supplied regarding data availability: The raw data are available in Table S1.
